# The immunoreceptor tyrosine-based activation motif (ITAM) -related factors are increased in synovial tissue and vasculature of rheumatoid arthritic joints

**DOI:** 10.1186/ar4088

**Published:** 2012-11-12

**Authors:** Tania N Crotti, Anak ASSK Dharmapatni, Ekram Alias, Andrew CW Zannettino, Malcolm D Smith, David R Haynes

**Affiliations:** 1 Discipline of Anatomy and Pathology, The University of Adelaide, Frome Rd., Adelaide, SA 5005, Australia; 2 Department of Biochemistry, Faculty of Medicine, National University of Malaysia, 50300 Kuala Lumpur, Malaysia; 3 Myeloma Research Laboratory, Centre for Cancer Biology, SA Pathology, Frome Rd, Adelaide, SA 5005, Australia; 4 Discipline of Physiology, The University of Adelaide, Frome Rd., Adelaide, SA 5005, Australia; 5 Department of Medicine, Flinders Medical Centre, Flinders Drive, Bedford Park, SA 5042, Australia; 6 Repatriation General Hospital, Daws Rd., Daw Park, SA 5041, Australia

## Abstract

**Introduction:**

The immunoreceptor tyrosine-based activation motif (ITAM) pathway provides osteoclast co-stimulatory signals and regulates proliferation, survival and differentiation of effector immune cells. In the osteoclast, the receptors Triggering Receptor Expressed on Myeloid cells 2 (TREM2) and Osteoclast Associated Receptor (OSCAR) and their respective adaptor proteins, DAP12 and FcRγ mediate ITAM signals and induce calcium signaling and the crucial transcription factor, NFATc1. In rheumatoid arthritis (RA), OSCAR expression by monocytes is inversely correlated with disease activity. Additionally, serum levels of OSCAR are reduced in RA patients versus healthy controls suggesting that expression and secretion or cleavage of soluble (s) OSCAR is immune modulated. Recent data suggest that endothelial cells may also be a source of OSCAR.

**Methods:**

ITAM receptors, their adaptor proteins, and NFATc1 and cathepsin K were detected in human synovial tissues by immunohistochemistry. Synovial tissues from patients with active RA were compared with tissue from patients in remission, osteoarthritis (OA) patients and healthy individuals. OSCAR was measured by immunoassay in synovial fluids recovered from active RA and OA patients. Endothelial cells were cultured with or without 5 ng/mL TNF-α or IL-1β over 72 hours. Temporal expression of OSCAR mRNA was assessed by qRT PCR and OSCAR protein in the supernatant was measured by ELISA.

**Results:**

Significantly higher (*P *< 0.05) NFATc1-positive inflammatory cell aggregates were found in active RA tissues than in healthy synovial tissue. Similarly, the percentage of OSCAR, FcRγ, DAP12 and TREM2 positive cells was significantly higher in active RA tissues compared to the healthy synovial tissue. Notably, OSCAR was strongly expressed in the microvasculature of the active RA tissues (9/9), inactive RA (8/9) weakly in OA (4/9) but only in the lumen of healthy synovial tissue (0/8). OSCAR levels were detected in synovial fluids from both RA (47 to 152 ng/mL) and OA (112 to 145 ng/mL) patients. Moreover, OSCAR mRNA expression and soluble OSCAR release was stimulated by TNF-α and IL1-β in cultured endothelial cells.

**Conclusions:**

Increased levels of ITAM related factors were present in synovial tissue from active RA joints compared to OA and healthy joints. OSCAR was strongly expressed by the vasculature of active RA patients and membrane bound and soluble OSCAR was stimulated by inflammatory mediators in endothelial cells *in vitro*.

## Introduction

Rheumatoid arthritis (RA) is an autoimmune disease that involves dysregulated immune cell functions. It is characterized by joint damage and systemic bone loss associated with excessive osteoclast activity [1-4]. Human studies show that mature osteoclasts are in close proximity to the bone surface in affected joints of patients with RA [2]. Identifying factors that regulate the differentiation and activity of osteoclasts is crucial in identifying potential targets to abrogate this local and systemic bone loss in RA.

A key molecule known to stimulate osteoclast differentiation and activity is receptor activator NF kappa B ligand (RANKL). Elevated RANKL in active RA relative to its inhibitor osteoprotegerin (OPG) is associated with increased osteoclast differentiation and resorption [5]. In active RA synovial tissue we, and others, have demonstrated that RANKL expression is significantly increased in lymphocytes and fibroblasts [6,7]. RANKL binds to its receptor, RANK, on osteoclast precursors instigating the differentiation of monocytes into multinucleated mature osteoclasts via activation of key signaling cascades involving the transcription factor, nuclear factor of activated T cells (NFATc1) (reviewed by Asagiri *et al. *[8]).

NFATc1 is not only crucial in the regulation of terminal osteoclast formation, but also plays a role in the immune system [8,9]. NFATc1 also regulates T cell differentiation and activation [10], such as that seen in inflammatory diseases such as RA. In osteoclasts, NFATc1 directly induces early and late stage osteoclast specific gene expression in the absence of RANKL [9,11-14]. To our knowledge the distribution of NFATc1 expression in synovial tissue from active RA joints has not been previously characterized.

Recent studies suggest RANK-RANKL induced osteoclastogenesis is enhanced by co-stimulatory signals mediated by immunoreceptor tyrosine-based activation motif (ITAM) harboring adaptors [15-17]. ITAM signalling is also involved in the regulation of effector immune cells proliferation, survival and differentiation [16,17]. DNAX-activating protein 12kDa (DAP12) and Fc receptor common γ chain (FcRγ) are similar ITAM-containing adaptor proteins relevant to osteoclast formation in physiological bone turnover [17-19]. In pre-osteoclasts and osteoclasts the ITAM adaptor proteins DAP12 and FcRγ associate with innate immune receptors; in particular, Triggering Receptor Expressed on Myeloid cells 2 (TREM2) [15,20-22] and Osteoclast Associated Receptor (OSCAR) [17,23], respectively, to activate calcium, induce NFATc1 and convey ITAM signaling [17].

Mice deficient in both *DAP12 *and *FcRγ *develop severe osteopetrosis [16] and exhibit an osteoclast multinucleation defect that is restored upon introduction of NFATc1 [17]. Evidence suggests DAP12 and TREM2 are required for differentiation and migration of osteoclasts as bone resorption is reduced in osteoclasts derived from mice with mutations in DAP12 and/or TREM2 *in vitro *[15,20,21]. In the context of RA, activation of macrophages by FcRγ is reported to induce cartilage destruction independent of inflammation [24]. It was thus suggested that interaction of FcRγ with immune complexes drives inflammation and induces bone loss indirectly [24]. We have recently demonstrated increased levels of OSCAR, FcRγ, TREM2 and DAP12 in peri-implant tissues [25]. To our knowledge the expression of FcRγ, DAP12 and TREM2 has not yet been demonstrated in human RA.

A single-nucleotide polymorphism within the promoter of the OSCAR gene has been linked to increased risk of postmenopausal osteoporosis [26]. In RA, OSCAR has been related to disease activity [27] and the potential of cells to differentiate into osteoclasts [28]. These findings support the contention that OSCAR plays a role in human bone turn over [26,28,29]. In osteoclasts the crucial transcription factor, NFATc1, induces OSCAR gene expression [30]. Furthermore, ligand-activated OSCAR interacts with FcRγ to produce an increase in intracellular calcium [31] that further stimulates NFATc1 expression. This establishes a positive feedback loop that results in marked elevation of both OSCAR and NFATc1 expression in terminal stages of osteoclast formation [8]. Administration of an OSCAR-Ig fusion protein inhibits osteoclastogenes *in vitro *[30,32]. These findings establish OSCAR as not only an important immune modulator but also a major player in the regulation of osteoclastogenesis.

OSCAR is expressed by osteoclasts as well as dendritic cells in humans and is involved in antigen presentation and activation of dendritic cells [33,34]. OSCAR has also been reported to be associated with osteoclasts near sites of erosion and the mononuclear cells adjacent to the microvasculature in RA patients [28]. Interestingly, more recent studies have identified OSCAR expression by endothelial cells [35]. This is similar to our observation that OPG expression and release by endothelial cells is regulated by inflammatory cytokines [36]. However, production and possible release of soluble OSCAR by endothelial cells in response to inflammatory cytokines present in arthritides has not yet been reported.

High levels of OSCAR have recently been demonstrated in the synovial tissues and monocytes isolated from RA patients, with these cells having a greater propensity for differentiation into osteoclasts [28]. Tumor necrosis factor (TNF)-α was found to induce OSCAR expression in monocytes isolated from RA patients [28]. However, serum levels of OSCAR were lower in RA patients compared with normal controls. These findings further suggest cell associated and soluble OSCAR is regulated by inflammatory cytokines that play a significant role in the pathogenesis of RA.

We aimed to investigate the distribution of ITAM receptors (OSCAR and TREM2) and their adaptor proteins (FcRγ and DAP12) in synovial tissues from patients with active RA (as yet untreated with disease modifying anti-rheumatic drugs (DMARDs) and inactive RA (patients in remission following treatment) compared with tissues from OA and healthy joints. We also aimed to assess whether OSCAR is detectable in synovial fluid from active RA and OA as its close proximity to the joint might better reflect localized disease activity. In addition to this, we sought to determine whether OSCAR is expressed and released by endothelial cells *in vitro *and whether this expression is regulated by inflammatory cytokines.

## Materials and methods

### Patient samples

Synovial tissue samples were obtained from the rheumatology unit in the Repatriation General Hospital, Daw Park, South Australia. RA patients fulfilled the American College of Rheumatology criteria for RA [37]. Active RA patients were yet to undergo DMARD treatments and had active joint inflammation while inactive RA patients were in remission after successful DMARD treatment and undergoing follow-up. A small-bore arthroscopy (2.7 mm arthroscope, Dyonics, Andover, MA, USA) was performed under local anesthesia, as previously described [38]. Biopsies of synovial tissues from RA patients were obtained from all accessible regions of the knee joint, but mainly from the suprapatellar pouch. OA samples were obtained at the time of knee replacement surgery and fulfilled published criteria [39]. Healthy samples were from patients attending a sports medicine clinic with unexplained knee pain at the time of a diagnostic arthroscopy [40]. Details of the patients and medication at the time of surgery are summarized in Table 1. The study protocol was approved by the institutional Medical Ethics Committee. Written informed consent was obtained from patients with diseased (OA, active and inactive RA) and healthy joints that were included in the study.

**Table 1:** Details of the patients and medication at the time of surgery

Groups	Active RA	Inactive RA	OA	Normal
Age (years)	62.5 ± 19.28	72.33 ± 7.07	69.22 ± 7.98	36.3 ± 10.39

Gender (male/total)	2/10	6/9	6/9	6/10

CRP (IU/mL)	83.90 ± 83.78	9.78 ± 8.21	NA	NA

RF (mg/L)	19.40 ± 44.10	1.44 ± 0.53	<20	NA

Erosion Positivity per total samples	2/10	2/9	0/9	0/9

DMARDS	NSAIDs 9 Prednisolone 1	SSZ 1 Im Gold 5 MTX 2 Plaquenil 1	No NSAIDs 5 Panadeine 1 NSAIDs 3	None 9 Allopurinol 1

CRP, C-reactive protein; DMARD, disease modifying antirheumatic drug; Im Gold, intramuscular sodium aurothiomalate; MTX, methotrexate; NSAIDs, non-steroidal anti-inflammatory drugs; RF, rheumatoid factor; SSZ, sulphasalazine

Tissues were fixed in 10% buffered formalin and embedded in paraffin. Five-micrometer sections were mounted on 3-aminopropyltriethoxy-silane (APTS) (Sigma, St. Louis, MO, USA) coated glass slides for hematoxylin and eosin (H&E) staining and assessment of tissue histology.

### Immunohistochemistry

#### Antibodies and reagents

Serial sections were stained with the following antibodies (Mab): mouse monoclonal IgG_1 _anti-NFATc1 ((clone 7A6, sc-7294) Santa Cruz Biotechnology, Santa Cruz, CA, USA) (4 μg/mL), goat polyclonal anti-human OSCAR (sc-34233, Santa Cruz Biotechnology) (10 μg/mL), rabbit polyclonal anti-human FC€R1G (LS-B2169, Lifespan Biosciences, Inc., Seattle, WA, USA) (1.25 μg/mL), rabbit polyclonal anti-human TREM2 (HPA010917, Sigma-Aldrich Pty. Ltd., Castle Hill, NSW, Australia) and rabbit polyclonal anti-human DAP12 (sc-20783, Santa Cruz Biotechnology) (2 μg/mL). A mouse monoclonal IgG_1 _anti-human cathepsin K (Cath K) (MAB3324 clone 182-12G5, Millipore (Billerica, MA, USA) (2 μg/mL) was used to detect the presence of any osteoclasts.

Secondary antibodies included 10 μg/mL polyclonal goat anti-mouse IgG (Dako Cytomation, Glostrup, Denmark), or 3 μg/mL goat anti-rabbit IgG (P0448, Dako, Glostrup, Denmark) or 7 μg/mL swine anti-goat IgG (ACI3404, Invitrogen Life Technology, CA, USA). Tertiary antibodies included 7 μg/mL swine anti-goat IgG (ACI3404, Invitrogen Life Technology) or 13 μg/mL rabbit anti-swine IgG (P0164, Dako, Glostrup, Denmark).

### Immunohistochemistry (IHC)

Sections were dewaxed and pre-treated with either 10 mM sodium citrate buffer pH 6.0 or 10 mM Tris-ethylenediamine-tetraacetic acid (EDTA) buffer pH 9.0 at 90 to 95°C for 10 to 20 minutes for antigen retrieval. Sections were treated with phosphate buffered saline (PBS)/0.1% sodium azide and 0.3% v/v hydrogen peroxide to inhibit endogenous peroxidase activity. A three-step peroxidase-based immunostaining technique, as previously described [41], with minor modifications, was employed. Sections were incubated with the primary antibodies (concentrations as described above) diluted in PBS and 1% bovine serum albumin (BSA) overnight at room temperature in a wet chamber. Sections were incubated with the appropriate HRP-conjugated secondary antibodies followed by incubation with the relevant HRP- conjugated tertiary swine anti-goat IgG or rabbit anti-swine IgG. HRP activity was detected using hydrogen peroxide as the substrate and 3-amino-9-ethylcarbazole (AEC) (K3469, Dako, **Carpinteria**, CA, USA) as the dye. Sections were counter-stained with Harris hematoxylin and lithium carbonate and mounted with GurrAquamount (British Drug House, Poole, UK). Negative controls included isotype-matched antibody controls (mouse IgG_1kappa _for mouse IgG_1_) and antibody-raised serum for polyclonal antibody (normal rabbit serum or goat serum) with equivalent concentration to the primary antibodies.

### Semi quantitative scoring analysis (SQA) of IHC result

Sections were scanned at high resolution using a NanoZoomer (Hamatsu, Shizouka, Japan), Digital Pathology, to enable quantification and archival of the IHC results. Three areas of 2 mm^2 ^were randomly selected and sections were assessed in random order by two blinded observers. Semi-quantitative assessment (SQA) of OSCAR, FcRγ, DAP12 and TREM2 and Cath K staining was scored using a 5-scale (0 to 4) scoring system [25,42]. Assessment was according to the percentage of positive stained cells as follows; 0 represented 0 to 5%, 1 for 6 to 10%, 2 for 11 to 25%, 3 indicated between 26 and 50% and a score of 4 indicated more than 50% of positive cells (adapted from [42]) within the sublining of the synovial tissue. For NFATc1 immunostaining, the number of positive stained cell aggregations (defined as more than 25 cells) was used as a parameter for grading (adapted from [43]). A score of 0 represented no positive stained cell aggregation, a score of 1 indicated one to three positive cell aggregations, score of 2 indicated total positive cell aggregation between four and six, seven to nine positive cell aggregations gave a score of 3 and a score of 4 indicated more than nine positive cell aggregations were present. Observations were made as to the presence of positive cells in the vessels or lining.

### Endothelial cell *in vitro *cultures

Bone marrow endothelial cells (BMEC) were cultured in triplicate in the presence and absence of 5 ng/mL TNF-α and IL1-β over a 72-hour time period. Expression of OSCAR and OPG mRNA levels by BMECs were assessed at 0, 6, 12, 24, 48 and 72 hours by quantitative reverse transcriptase polymerase chain reaction (QRT PCR). Only data from 0, 24, 48 and 72 are presented here. Supernatants were collected at 0, 24, 48 and 72 hours to assess soluble OSCAR and were stored at -20°C until use in ELISA assay. BMECs were also cultured on chamber slides for immunofluorescent detection of OSCAR and OPG.

### ELISA analysis of soluble OSCAR

Synovial fluid was obtained from patients at the rheumatology unit in the Repatriation General Hospital, Daw Park, South Australia. The patient cohort consisted of synovial fluid from active RA (average age 67, CRP 67.4 IU/mL and RF 165 mg/L, 7 male/5 female, 4/12 with erosion) and OA joints (average age 70, 3 male/5 female). Levels of OSCAR were assessed by ELISA kit following manufacturer's instructions (USCN Life Science, Inc., Wuhan, China). Samples (synovial fluids and supernatants) were clarified by centrifugation at 13,000 rpm for five minutes at 4°C. The supernatant was transferred to 1.5 mL eppendorf tubes and diluted 1/5 for synovial fluid and 1/10 for supernatant in the sample diluent provided in the kit. One hundred microliters of pre-diluted samples were loaded into each well along with the protein standards provided. Assays were carried out in duplicate and the OSCAR protein concentration in each sample was determined based on the standard curve generated.

### RNA extraction and cDNA synthesis

Total RNA was isolated from *in vitro *BMEC cultures following the addition of 500 μL TRIzol reagent per well, as per the manufacturer's instructions (Invitrogen Life Technologies, Carlsbad, CA, USA). Complementary DNA (cDNA) was synthesized from 1 μg RNA per reaction using Superscript III Reverse Transcriptase (Invitrogen Life Technologies), as previously described [44].

### Quantitative real time reverse-transcription polymerase chain reaction (QRT-PCR)

Real-time PCR was performed using Platinum SYBR Green qPCRSupermix-UDG (Invitrogen Life Technologies), as per the manufacturer's recommendations. Amplification was carried out in a Rotor-Gene 3000. Reaction mixtures contained 1 μl of 1 in 5 pre-diluted cDNA, 7.5 μl Platinum SYBR Green qPCR Supermix-UDG, 300 nM each of forward and reverse primers and diethyl pyrocarbonate (DEPC)-treated water to a final volume of 15 μl. Primer3Plus freeware [45] was used to design oligonucleotide primers to human OSCAR Forward 'CCC AGC TTC ATA CCA CCC TA' and Reverse: 'GAA GAG AAG GGG AGC GAT CT' [46]. Primer sequences for OPG and the endogenous reference gene glyceraldehyde 3-phosphate dehydrogenase (GAPDH) were designed as described previously [47]. All samples were investigated in triplicate QRTPCR reactions. Fold induction was calculated as a measure of 2^ddCT ^[48].

### Immunofluoresence of BMEC

BMECs were grown for 40 hours in the absence or presence of 5 ng/mL IL-1β or TNF-α. Cells were fixed with 1:1 methanol:acetone for five minutes and washed with PBS. Primary antibodies OPG MAB805 (10 μg/mL) or polyclonal goat anti-human OSCAR (8 μg/mL, sc-34233, Santa Cruz Biotechnology) were diluted in PBS with 1% BSA and incubated overnight at room temperature. To control for non-specific staining of the OSCAR antibody, wells were incubated with normal goat serum in the absence of the antibody to OSCAR. Following washing with PBS, OSCAR and OPG antibodies were detected with secondary antibodies to goat conjugated with FITC (green) and mouse conjugated to cy3 (red) (both from Southern Biotechnology Associates, Inc., Birmingham, AL, USA), respectively, diluted in PBS plus 1% BSA for 30 minutes then washed three times with PBS.

### Statistical analysis

Power calculations demonstrate sufficient power to detect differences between the healthy subjects and joints with active RA for each of the molecules detected (92.9 to 100%). To assess the SQAs assigned by analysis of the IHC staining statistical analysis was performed using SPSS version 11.5 **(SPSS Inc**, Chicago, IL, USA). A non-parametric Kruskal-Wallis analysis was used to compare the mean of the SQA score between the groups. A Mann-Whitney-U test was used to examine the significant difference between two groups, with a *P-*value <0.05 accepted as statistically significant.

Differences in the soluble OSCAR levels in synovial fluids between the two groups were analyzed by Student's *t-*test and *P *< 0.05 was considered significant. Statistical significance between treatments and time points was determined using Kruskal-Wallis followed by the Mann-Whitney test using GraphPad Prism version 5.0d (GraphPad Software Inc. La Jolla, CA, USA).

Differences in mRNA levels between the groups at each time point were analyzed using a Two-way Anova test.

## Results

### Expression of ITAM modulatory factors in active RA

**Figure 1:**
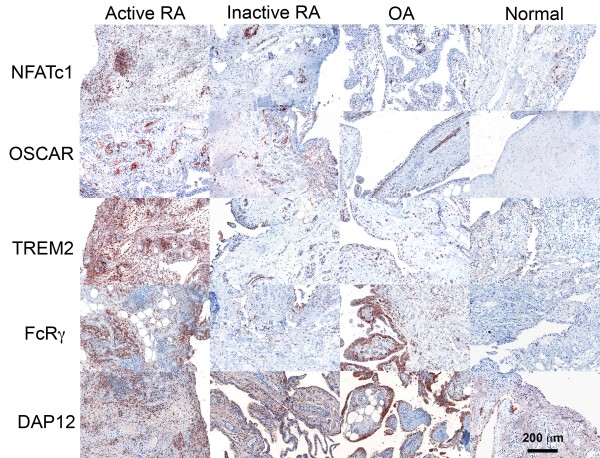
**Expression of NFATc1 and ITAM associated molecules in human synovial tissues**. Detection of factors was visualised with AEC (red) and counterstained with heamatoxilyn (blue) 100X mag.

While NFATc1 expression was localized in isolated cells throughout the tissue, NFATc1 was mainly expressed by cells within lymphocyte aggregates in tissues from untreated active RA joints (Figure 1). The SQA grading for NFATc1 immunostaining was, therefore, based on the number of cell aggregates expressing NFATc1 (described in Methods). The number of NFATc1-positive cell aggregations was found to be significantly more in the active RA group compared to all other groups (*P *< 0.05) (Table 2).

**Table 2:** Semi-quantitative analysis of staining for NFATc1 and ITAM factors within tissue

	Act RA	Inact RA	OA	Norm
**NFATc1**	2.50 ± 0.21 ♦‡◘ (*n *= 10)	0.75 ± 0.23 (*n *= 8)	0.77 ± 0.24 (*n *= 9)	0.28 ± 0.21 (*n *= 7)

**Cath K**	0.6 ± 0.22 (*n *= 10)	1.12 ± 0.35 (*n *= 8)	1.33 ± 0.41 (*n *= 9)	1.89 ± 0.35 (*n *= 9)

**OSCAR**	1.44 ± 0.24 (*n *= 9)	0.89 ± 0.39 (*n *= 9)	0.89 ± 0.26 (*n *= 9)	0 ± 0 (*n *= 8)

**TREM2**	3.44 ± 0.17♦‡◘ (*n *= 9)	2.55 ± 0.34◘ (*n *= 9)	2.44 ± 0.24◘ (*n *= 9)	1.77 ± 0.22 (*n *= 9)

**FcRγ**	2.50 ± 0.31 ♦◘ (*n *= 10)	0.88 ± 0.40 (*n *= 8)	2.22 ± 0.40 ♦◘ (*n *= 9)	0.70 ± 0.26 (*n *= 10)

**DAP12**	1.70 ± 0.21♦‡◘ (*n *= 10)	0.67 ± 0.29 (*n *= 9)	0.89 ± 0.11◘ (*n *= 9)	0.40 ± 0.22 (*n *= 10)

Mean Score ± SEM; ♦ *P *< 0.05 significantly different from Inactive RA, ‡ *P *< 0.05 significantly different from OA, ◘ *P *< 0.05 significantly different from Normal.

Cathepsin K (Cath K) is routinely used as a marker of osteoclast-like cells as it is expressed at high levels in osteoclasts [49,50] and is the main bone-matrix degrading enzyme [51,52]. Immunostaining for Cath K was found to be predominantly in the sublining of the synovium and expressed by synovial fibroblasts (images not shown). The proportion of Cath K positive cells was not significantly different between groups.

**Figure 2:**
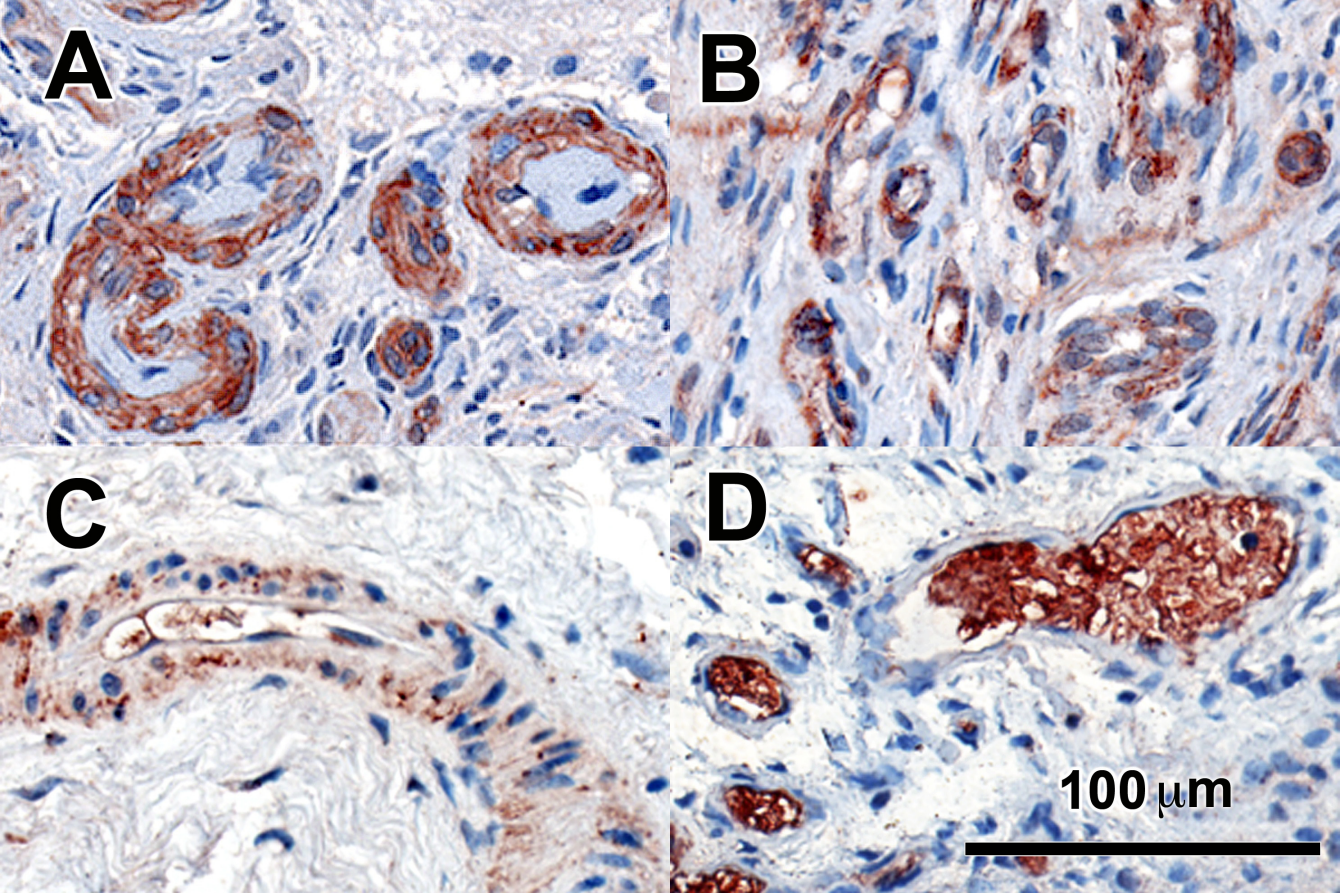
**OSCAR staining in associated with the vasculature in active (**A**) and inactive RA (**B**) synovial tissues as well as OA (**C**) and normal synovial tissues (**D**) (red stain) at 200X mag**.

OSCAR has been reported to be increased in RA compared to that in OA tissues [28]. OSCAR expression was associated with macrophage-like cells in the sublining of the synovium of inactive and active RA patients as well as in the lining of OA patients (Figure 1). Strongly positive OSCAR staining was associated with the microvasculature of the synovium in patients with active RA, nine of nine (Figure 2A) and with inactive RA (eight of nine) (Figure 2B). OSCAR was weakly expressed in four out of eight of the tissue samples from OA patients (Figure 2C) and was absent in the microvasculature of the healthy tissues (zero of nine) (Figure 2D). Interestingly, OSCAR appeared in the lumen of the microvasculature of the synovium from healthy patients that was suggestive of cleaved or soluble protein in the serum.

TREM2 was highly expressed throughout tissue from active RA patients (Figure 1). Many types of cells appeared to express TREM2, including mononuclear cells in lymphoid aggregates and fibroblasts with expression significantly greater than all other groups (Table 2). TREM2 expression was also associated with the microvasculature of active and inactive RA patients.

FcRγ was also highly expressed in active RA tissues and OA patient tissues (Figure 1). This was significantly higher in comparison to the inactive RA and the control tissues (*P *< 0.05) (Table 2). FcRγ was strongly expressed in macrophage-like synovial lining cells particularly in the OA patients. In active RA, FcRγ appeared to be expressed by macrophage and fibroblast-like cells throughout the tissue but was absent in the lymphoid aggregates and not associated with the microvasculature.

DAP12 expression was significantly higher in active RA compared with all other groups (*P *< 0.05) (Table 2). DAP12 appeared predominantly associated with macrophage-like cells in the sublining of the synovial tissue and the macrophage-like lining cells of the OA group (Figure 1). Of note, the microvasculature was negative in all groups.

### Soluble OSCAR in OA and RA synovial fluid

**Figure 3:**
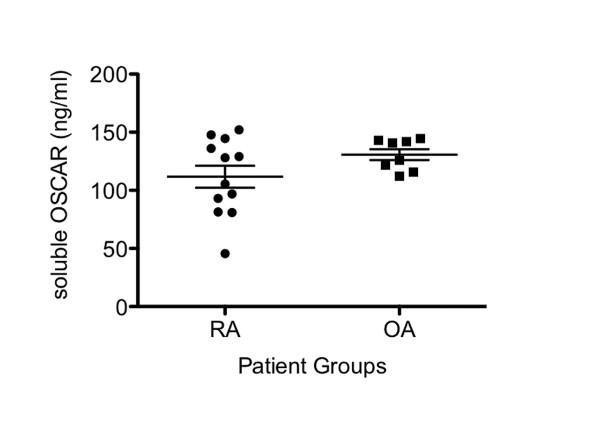
**Levels of soluble OSCAR in synovial fluids from RA and OA patients as measured by ELISA**. Each point represents levels in an individual patient.

Soluble OSCAR was detected in serum from RA patients and normal individuals by Herman [28], suggesting a cleaved or released form of OSCAR. To more closely assess OSCAR in relation to disease activity near the joint, we measured soluble OSCAR in the synovial fluid from OA (average age 70, 3 male/5 female) and RA patients (average age 67, 7 male/5 female). We detected soluble OSCAR in OA, (112 to 145 ng/mL) with more variable levels in active RA groups (47 to 152 ng/mL) and no significant difference between the groups (Figure 3).

### OSCAR expression in BMECs

**Figure 4:**
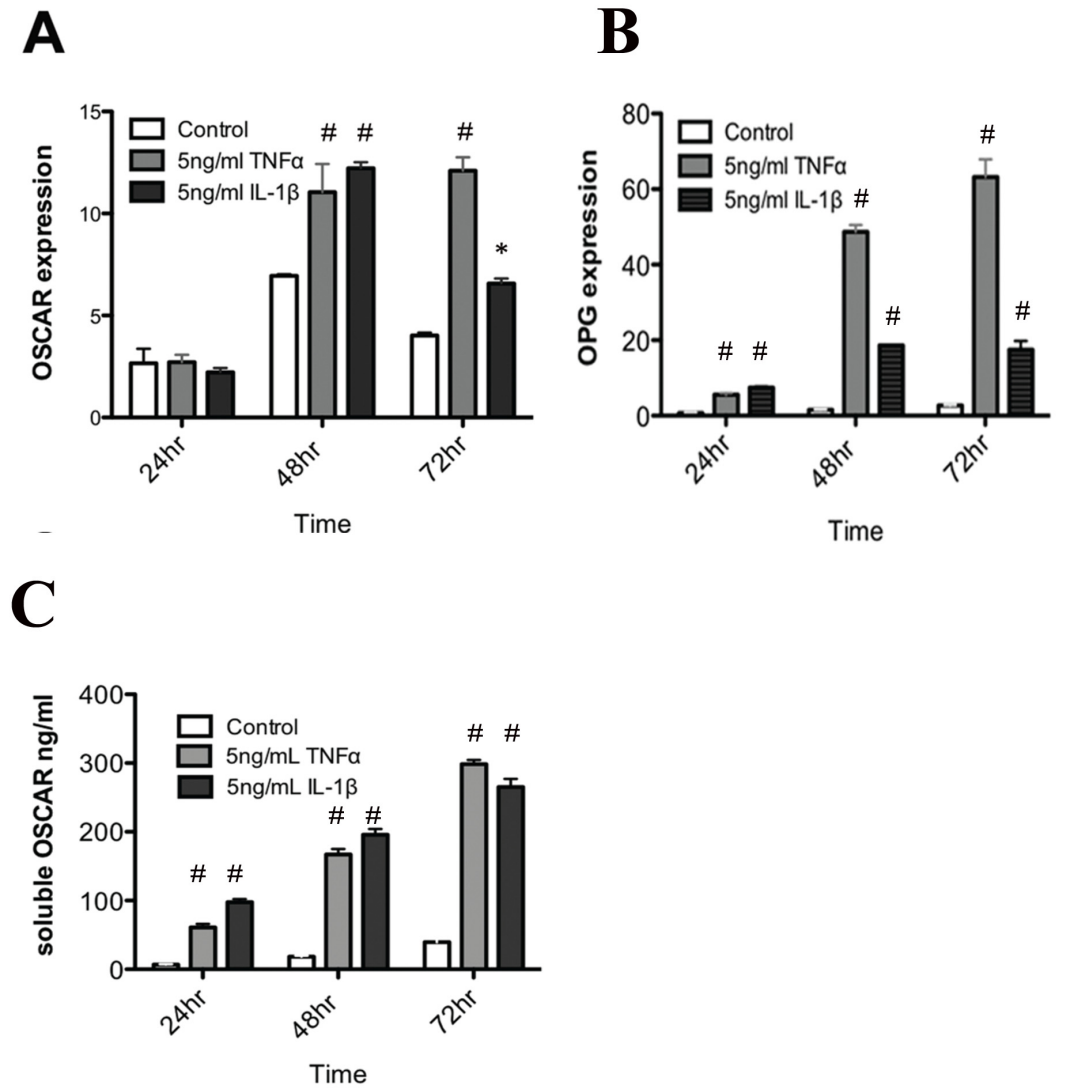
**Expression of OSCAR by endothelial cells in response to inflammatory cytokines**. BMECs were cultured in triplicate in the presence or absence of recombinant TNF-α and IL1-β. **A**. OSCAR gene expression was assessed at 0, 24, 48 and 72 hours by QRT PCR. GAPDH was used as the housekeeping gene. Fold induction was calculated as a measure of 2^ddCT^. **B**. Induction of OPG gene expression at 0, 24, 48 and 72 hours. **C**. Release of soluble OSCAR was assessed in duplicate, by ELISA, as per the manufacturer's instructions. The mean values were calculated. Significance of *P *< 0.05 (*) and *P *< 0.001 (#) are indicated.

**Figure 5:**
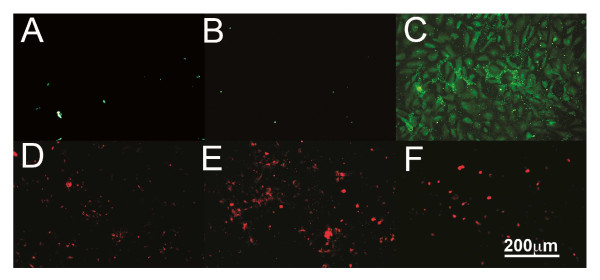
**OSCAR and OPG expression by BMECs**. BMECs were cultured in the absence (**A **and **D**) or presence of 5 ng/mL IL-1β, (**B**, **E**) and 5 ng/mL TNF-α (**C**, **F**) for 40 hours and fixed for visualization by con-focal microscopy. OSCAR is seen as green (A, B, C) and OPG as red (D, E, F). Original magnification was 200X.

OSCAR expression by human umbilical vein endothelial cells (HUVEC) has been recently reported [35]. In our IHC analysis, we observed high levels of OSCAR associated with the microvasculature in the sublining region of synovial tissue from active and inactive RA synovial tissue joints (Figure 2A, B), with low levels associated with OA microvasculature (Figure 2C). It was interesting to observe that OSCAR was present in the lumen of the microvasculature in the synovium of normal patients (Figure 2D). These findings suggest soluble OSCAR may be present that is likely mediated by inflammatory cytokines present in RA tissue [53]. In view of this, we assessed whether OSCAR mRNA expression and release of soluble OSCAR by endothelial cells was modulated by inflammatory mediators associated with RA. OSCAR mRNA was significantly increased in cultured endothelial cells by IL-1β at 48 and 72 hours (*P *< 0.05 and 0.001 respectively) and TNF-α at 48 and 72 hours (*P *0.001) compared with untreated cells at these time points (Figure 4A). Similarly, TNF-α and IL-1β significantly increased OPG expression by BMECs both at 48 and 72 hours (*P *< 0.001), consistent with our previous report using HUVEC cells [36] (Figure 5B). Based on these findings, supernatants from the cytokine stimulated BMEC cultures were assessed for OSCAR protein after 24, 48 and 72 hours of treatments (Figure 4C). We found increasing levels of soluble OSCAR over a 72-hour time period in response to both TNF-α and IL-1β with *P *< 0.001 at all time points compared with untreated BMECs.

### Confocal detection of OSCAR in BMECs

To ascertain whether cell bound OSCAR is regulated by inflammatory cytokines, OSCAR was detected by immunofluorescence in cytokine stimulated BMECs. This was compared to OPG in the light of our previous findings that showed OPG is up-regulated by TNF-α in endothelial cells [36]. Following IL-1β treatment, little OSCAR but higher levels of OPG were detected (Figure 5B, E, respectively). TNF-α induced strong protein expression of OSCAR and little OPG expression. OSCAR appeared to be present in vesicles as there was strong punctate staining as well as diffuse cellular staining (Figure 5C). In all the treatments, the pattern of OSCAR expression was distinctly different from that of OPG.

## Discussion

ITAM-related molecules are likely to be important mediators of RA joint destruction through their regulation of immune-mediated inflammation and bone erosion by osteoclasts [29]. In osteoclasts, the ITAM co-stimulatory pathways activate calcium and induce NFATc1 in pre-osteoclasts [17] to further enhance osteoclast differentiation and activation [9]. NFATc1 in activated T-cells regulates their differentiation and activation [10] and may further stimulate osteoclastogenesis via stimulating RANKL expression [54]. We observed significantly increased aggregates of NFATc1 positive cells in active RA synovial tissue. These are likely to represent populations of activated T-cells in the rheumatoid tissues. While low numbers of NFATc1 positive cells with the morphology of multinucleated pre-osteoclasts were observed in the current study, this may be due to the tissue sections not including juxtaposing bone where later stage active osteoclasts would be present [55]. Furthermore, our samples consisted of synovial tissue rather than pannus where osteoclast precursors are higher in number [2]. For similar reasons we did not note a significant increase in osteoclast-like cells expressing Cath K in active RA synovial tissue. Additionally, we observed Cath K associated with fibroblasts, consistent with published reports describing Cath K expression in skin fibroblasts [56,57]

Consistent with the findings reported here, TREM2 has been reported as expressed by macrophages, osteoclasts and, more recently, endothelial cells [15,22,58]. The ITAM-containing adapter molecule, DAP12, associates with TREM2 receptors in a number of cell types, including osteoclasts [15,20,22,59]. DAP12/TREM2 signaling has been shown to regulate inflammatory responses [59], play an important role in antigen presentation by dendritic cells [20] and has been implicated in T cell regulatory activity [60]. We observed markedly higher levels of DAP12 and TREM2 in active RA patients. Multiple cell types expressed TREM2, including mononuclear cells in lymphoid aggregates and fibroblasts. TREM2 expression was also associated with the microvasculature of active and inactive RA patients. Interestingly, DAP12 appeared predominantly associated with macrophage-like cells in the sublining of the synovial tissue, particularly in the macrophage-like cells in the lining of the OA group.

OSCAR and FcRγ form an ITAM receptor/signaling pair that is expressed in osteoclasts and transmits ITAM signaling to induce osteoclast differentiation [16,23]. We observed high levels of FcRγ in association with fibroblasts and monocytes of the synovial sublining while lymphoid aggregates and the vasculature did not express FcRγ. Of note, similar to DAP12, FcRγ was associated with macrophage-like synoviocytes in the synovial lining with some scattered monocytes in the sublining of the OA tissue. The increased DAP12 and FcRγ expression may indicate a role in the pathogenesis of OA but this is yet to be determined.

OSCAR is a functional receptor on monocytes and neutrophils involved in the induction of the primary pro-inflammatory cascade and the initiation of downstream immune responses [31]. Ligation of human OSCAR on monocytes and neutrophils also results in the induction of a pro-inflammatory cascade and downstream immune responses [31]. High levels of OSCAR have recently been demonstrated in the tissues of RA patients and the serum of healthy individuals [28]. This is consistent with our findings that OSCAR protein was increased on monocytes from RA patients compared with healthy individuals with expression correlating with inflammatory disease activity [28].

Interestingly, OSCAR expressing monocytes were seen adjacent to microvasculature, consistent with observations by Herman *et al. *[28]. In a previous study, the expression of OSCAR was shown in multinucleated osteoclast-like cells attached to the bone [28]. We could not confirm this as the synovial tissue specimens from the RA patients used in this study did not include bone [55,61]. However, we did detect positive macrophage-like cells expressing OSCAR in the lining of the OA specimens similar to that previously reported [28]. Herman and colleagues have suggested that OSCAR expression might be regulated by pro-inflammatory cytokines [28]. Therefore, increased expression of OSCAR in active RA synovia compared to other groups is likely to be due to elevated inflammatory cytokines present in RA synovial tissues.

A possible limitation of the study is that both groups were not perfectly age- and sex-matched. It is very difficult to age- and sex-match the RA, OA and normal tissues due to the nature of the joint diseases affecting predominantly different age groups and sexes. In addition, patients presenting with active RA can take several years of treatment before the disease is in remission and classified as inactive. While to our knowledge there is are no reports that OSCAR expression changes with age, it is possible the differences in age may influence the results of our study.

It is possible that soluble OSCAR may act as a decoy receptor and suppress ITAM signaling. Of particular interest is the observation that RA patients had reduced levels of soluble OSCAR in serum compared to healthy individuals [28]. However, we noted high levels of OSCAR in the synovial fluid of our active RA patients and OA patients. A possible reason for this is that in the Herman *et al. *study patients with RA were being treated with anti-TNF therapy, unlike our active RA patients. It is also possible that levels in the joint locally are not necessarily reflected systemically in the circulation. The lack of significant differences between soluble OSCAR in OA and RA patients may be due to the low sample number or may reflect the increased release of OSCAR in response to inflammatory cytokines present in both of these pathologies [38,53].

High levels of OSCAR were associated with the microvasculature of the synovium of active RA specimens as well as tissues taken from our inactive RA patient group, who have had successful DMARD treatments. In the synovial tissue of healthy joints, positive OSCAR staining was present only in the lumen of the vasculature, suggesting the presence of soluble OSCAR in the blood. These observations were consistent with the recent finding that human primary endothelial cells express OSCAR [35]. Contrary to our findings, Herman *et al. *did not observe OSCAR staining of the microvasculature of his RA patients. This again may be due to the fact that, unlike our active RA patients, the patients in the Herman study were being treated with anti-TNF therapy.

The increased expression of OSCAR associated with the microvasculature in RA compared with normal tissue suggests that OSCAR expression is modulated by immune mediators or cytokines. Our previous studies have shown a reverse pattern of OPG expression in the vasculature, with low OPG levels associated with RA, compared with high levels associated with normal tissue [41]. Additionally, our *in vitro *studies showed that OPG expression in HUVEC is regulated by cytokines [36]. Our *in vitro *studies extend the findings by Goettsch *et al. *[35] and demonstrate that the expression of OSCAR by endothelial cells is stimulated by the inflammatory cytokines, TNF-α and IL-β, and is consistent with our *in vivo *findings showing OSCAR expression was elevated in the untreated RA synovial vasculature. Importantly, endothelial cells are a likely source of OSCAR and not just binding soluble OSCAR present in the serum, as we demonstrated that the inflammatory cytokines stimulated both mRNA and protein *in vitro*. TNF-α also induces OSCAR expression in monocytes *in vitro *[28]. This indicates that soluble OSCAR in the serum [28] and synovial fluid in RA is released by a variety of cells and is stimulated by inflammatory cytokines.

As recombinant human OSCAR-Fc is able to act as a decoy receptor for cell bound OSCAR [28], soluble OSCAR might provide a protective mechanism against bone erosion *in vivo *by competing with a ligand for cellular OSCAR and reducing signaling for inflammatory cells and osteoclasts. We propose that successful treatment of RA results in increased cleavage of OSCAR resulting in increased soluble OSCAR levels [27,28]. This is consistent with the observation that patients in remission have higher levels of soluble OSCAR and suggests a role in modulating osteoclastic bone resorption.

While we were able to demonstrate OSCAR expression in RA and OA synovial fluids for the first time, our data on the relative levels suggest that it is not a good discriminator between RA and OA. The data of Herman [28] and Zhao [27], who measured blood levels, suggest blood levels are lower in active RA compared to inactive disease. However, induction of OSCAR release *in vitro *and the levels of expression in the tissue indicate its expression, at least locally, may be related to disease activity. Further studies following patients during treatment may help resolve whether it is a marker for assessing disease activity and joint erosion in RA.

## Conclusions

Here we present evidence that NFATc1 and the ITAM-related molecules FcRγ, TREM2, DAP12 and NFATc1 are up-regulated in active RA synovia in comparison to healthy control or inactive RA and OA tissues. In addition to this, we find higher proportions of OSCAR positive vessels in both active RA patients and inactive RA patients compared with healthy controls. Our *in vitro *studies also confirm OSCAR expression by endothelial cells and demonstrate regulation of production and release of soluble protein by inflammatory cytokines, IL-1β and TNF-α. Of interest, we were able to demonstrate OSCAR expression in RA and OA synovial fluids for the first time.

## Abbreviations

AEC: 3-amino-9-ethylcarbazole; APTS: 3-aminopropyltriethoxy-silane; BMEC: bone marrow endothelial cells; BSA: bovine serum albumin; Cath K: cathepsin K; DAP12: DNAX-activating protein 12kDa; DEPC: diethyl pyrocarbonate; DMARDs: disease-modifying antirheumatic drugs; EDTA: ethylenediamine-tetraacetic acid; FcRγ: Fc receptor common γ chain; GAPDH: glyceraldehyde 3-phosphate dehydrogenase; H&E: hematoxylin and eosin; HRP: horse-radish peroxidase; HUVEC: human umbilical vein endothelial cells; IHC: immunohistochemistry; ITAM: immunoreceptor tyrosine-based activation motif; NFATc1: nuclear factor of activated T cells; OA: osteoarthritis; OPG: osteoprotegerin; OSCAR: osteoclast associated receptor; PBS: phosphate-buffered saline; RA: rheumatoid arthritis; RANKL: receptor activator NFkappa B ligand; SQA: semi-quantitative assessment; TREM2: triggering receptor expressed on myeloid cells 2.

## Competing interests

The authors declare that they have no competing interests.

## Authors' contributions

TC, EA and AD made substantial contributions to conception of the study, the design of experiments, the acquisition of data, data analysis and interpretation of data. TC, EA, AD, AZ MS and DH made substantial contributions to conception of the study, the interpretation of data and were involved in drafting the manuscript. All authors have read and approved the manuscript for publication.
